# Patient-Related Complexity of Care in Healthcare Organizations: A Management and Evaluation Model

**DOI:** 10.3390/ijerph17103463

**Published:** 2020-05-15

**Authors:** Fiorella Pia Salvatore, Simone Fanelli

**Affiliations:** 1 Department of Economics, University of Foggia, 71121 Foggia, Italy; 2 Department of Economics and Management, University of Parma, 43125 Parma, Italy; simone.fanelli@unipr.it

**Keywords:** healthcare organization, health management, Italian health system, patients allocation model, ICC

## Abstract

In times of economic stringency, the prerequisite for the provision of healthcare services differentiated by complexity is identified in the right patients’ allocation. Since access to high-intensity care units is restricted, it is necessary both to promptly diagnose patients who are at risk of rapid clinical deterioration or death and to define criteria to identify the correct allocation of patients based on clinical-care needs. Although the so-called “early warning scores” were used by healthcare professionals to alert medical staff, nowadays, they can also be used as decision rules for managing patient admissions, increasing their effective usefulness. The procedure for assessing the complexity of care profiles needs to be based on a multidisciplinary approach. The primary objective of scientific research was to determine the intensity of care (clinical instability and care dependence) of the patients allocated in different settings of the medical area. To correctly frame the phenomenon, the main methods and strategies developed for different care models were discussed. In the Italian healthcare organization, the indicators, methodologies and tools to evaluate the clinical-care complexity were identified and subsequently applied. In conclusion, the findings and proposals for improvement actions are shown.

## 1. Introduction

In a constantly evolving social, demographic and epidemiological context, the healthcare organization system should reconfigure itself to provide adequate answers to new care public needs [1]. Specifically, the changing path must take place on the basis of some essential points: a continuous review of the organizational processes of clinical governance, a progressive improvement of general health and a progressive improvement of the quality of services [2,3].

Within the Italian system, the issue of defining new organizational models for healthcare service drew the attention of the legislator, and in recent years, regional legislative interventions have contributed to further fueling the already heated scientific and political debate [4,5]. For example, the 40/2005 Regional Disposition (regional health system’s reform) of the Tuscany Region, in order to achieve the objective of promoting the efficiency of hospital activities, has identified three specific guidelines:

- Definition of differentiated areas, based on the assistance modalities;
- Structuring by caring complexity;
- Gradual overcoming of the articulation by Operating Units.

Based on these assumptions, the Tuscany Region has identified innovative experiences consolidated in various areas [6]:

- Operating theatre re-modulation. The hospitalization duration considered as a focal point has allowed introducing a new way to classify the hospitalizations. The average hospitalization duration can be used as an indicator of complexity, in order to reserve a part of the interventions and hospital beds for the cases resolvable within four days of the intervention (week surgery) and allowing its closure on the weekend [7];
- Modular-care model. It was born in a nursing team, in which the key figure is the nurse who plans, implements and supervises the care of assigned patients, collaborating with other professional healthcare figures [8];
- Care complexity model. It aimed at responding to the need to increase both the complexity of patients with high degrees of clinical instability level and the increase in the care demand due to the significant presence of elderly patients with comorbidities [9].

To date, there is no complete agreement on the model to be aimed at, but a good healthcare system economically sustainable and compatible with the clinical efficacy principles should support the healthcare organizations and include at least the following organizational changes [10,11]:

- A new work organization allowing to take charge of the patient and his/her care path efficiently through the development of the digital medical record;
- A new operating unit organization, characterized by greater flexibility obtained through the maximum integration of skills, adequate levels of care and optimized volumes of activity;
- A new hospital flow organization envisaging an emergency classification and an improved flow organization, by complexity and care duration.

By making these changes, the healthcare system will be able to ensure effective and efficient responses to multi-professional organizational needs that gradually advance towards an increasingly user-centered healthcare model.

In the various healthcare contexts, the basic organizational model for the care complexity has changed in order to adapt to the different local situations, slightly modifying with respect to the fundamental principles. In particular, the concept of care dependence has not found a clear definition yet, but in the literature the most widespread formulation is the one offered by Moiset [12], which defines care dependency as:

“The set of interventions carried out by nursing care and expressed in terms of the intensity and quantity of activities of the healthcare professional.”

Instead, care complexity means the clinically required complexity taking into account the patient’s pathology and the specific alterations of the physiological parameters [13]. The most quoted definition is [14] as follows:

“Assign the patient to the most appropriate operating unit considering his care needs related not only to the hospitalization type but also to his clinical condition and care dependence.”

Considering the technologies, the skills and the type of staff, four levels of care complexity are identified [3,15]:

- Sub-intensive;
- High care;
- Medium care (ordinary hospitalization and short-cycle hospitalization);
- Low care (rehabilitation, post-acute care).

A FADOI [16] position statement highlights that the current healthcare’ organization is based on the traditional division into medical areas and operating theatres and, within them, into operating units. This implies that an extreme variability of clinical severity and care’s load represents a usual situation. A healthcare standard may be higher than the needs for some patients but insufficient for other patients. The consequences of this ‘‘flattening to the average’’ are known respectively as the “roof” and the ‘‘floor” effect. The “roof” effect occurs when a user with high needs is placed in a "low supply system" and tends to stress the system by obtaining more assistance than other hospitalized users without ever receiving the necessary one, resulting in inadequate assistance.

**Figure 1:**
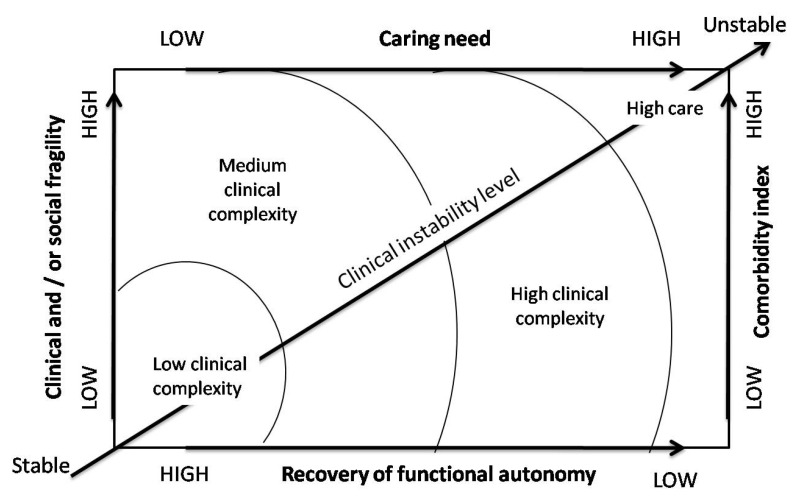
Model of the variables that make up the clinical care complexity. Source: Nardi et al. [17].

The “floor” effect occurs when a user with modest needs is inserted in a “high-offer system” receiving a higher share of assistance than is necessary; both from a quantitative and qualitative point of view, there is a waste of resources. The operative part of the study is represented in Figure 1 which shows the correct balance between floor and roof effects.

The following section describes the methods and tools for evaluating clinical-care complexity. The Italian organizational context and the indicators applied are shown later. The results and improvement proposals are shown in the conclusion.

### Methodologies for the Evaluation of Clinical Care Complexity: National and International Review

To evaluate the patient’s care dependence, there are several methods developed in relation to specific situations. These methods identified in the literature complicate the definition for universally accepted and standardized parameters. According to Cavaliere [18], the criteria adopted to measure the patient’s care dependence can be traced back to three main macro-areas. These are classified by:

- Documentation’s identification of the activities carried out;
- Patient’s profile identification;
- Assistance indicators’ identification.

The first area, documentation’s identification of the activities, provides a list of the tasks performed by the health professionals and defines their duration. The second area identifies the categories of patients considering specific characteristics from the care needs’ point of view. The healthcare professional’s workload, being dependent on the number of patients attributed, can be predetermined and standardized on the basis of the activity data of each setting. Instead, the methodologies for measuring care dependence require the recognition of the patient’s specific needs deriving from both care needs and clinical conditions. These indicators are assigned a numerical score and the workload of the health professional is determined by the sum of the scores of the individual indicators [18].

Through various methodological approaches, numerous tools have been internationally developed to measure human resources efficiency. The level of healthcare assistance can be defined through 3 criteria:

- Criterion based on the indications contained in the assistance plans, on the documentation of the activities carried out and on the quantification of the need for assistance in terms of "time" (for example, Project Research of Nursing);
- Criterion based on the patient’s global need (for example, the Swiss Method);
- Criterion based on care dependency and indicators (for example, Patient Intensity for Nursing Index method).

The main characteristics are summarized below.

Project Research of Nursing (PRN): The PRN [12] was developed in 1969 in Canada and is based on the quantification of the healthcare need in terms of “time spent for healthcare”. In order to define the care-weight of patients, based on the time spent for the various healthcare activities, the PNR identifies a plan with the healthcare activities to be implemented in the next twenty-four hours after admission to the hospital. The plan initially formulated is reviewed daily by the healthcare team until the patient’s discharge. Each healthcare action is assigned one or more points (based on the patient’s autonomy, the number of professionals involved, etc.) and each point equals five minutes of work by the healthcare professional. The method’s limit is identified in the time required for the application and in the complexity of the calculation.

Swiss method: Based on the identification of the global need for patient assistance, the Swiss method aims to classify patients into addictive classes. This method can be used both when evaluating the patient and when planning activities. Care assistance is closely linked to the degree of patient dependence and grows proportionally [19].

The healthcare activities are divided into three groups: (a) direct care, indicates the basic direct care activities included in the staff functions; (b) indirect care, indicates the activities connected with the management of the nursing staff; and (c) board activities, indicates the activities of the support staff centered on the service provision but different from the healthcare provision.

The patients’ classification into three categories is obtained by filling in a form containing a series of criteria to express the healthcare needs of the hospitalized individual person. Once the form has been completed, the final markings of each column are counted separately for the three sections. The section with the most markings determines the patient’s classification in the specific category.

Patient Intensity for Nursing Index (PINI): The PINI [20] is useful for defining the healthcare dependence. This method takes into consideration four conceptual dimensions: (a) pathology severity degree; (b) patient dependence degree; (c) complexity of care activities; (d) time spent. These conceptual dimensions are defined by 10 items, evaluated with an ordinal scale of 5 points. The severity of the disease is a concept related to the severity of the patient’s clinical condition; patients with the same pathology may be in profoundly different clinical stability–instability conditions.

In Italy, experiments to both adopt scientific tools for the healthcare evaluation and to identify a language shared on a professional level have recently developed. The characteristics of the 3 main tools developed in Italy are summarized below.

Metodo Assistenziale Professionalizzante (MAP): MAP procedure [21] was created in 2007 on the basis of the "Complexity Theory" and the "Model of Care Complexity Analysis". MAP includes two tools: the first allows to evaluate the care dependency of the patient, while the second one estimates the need for human resources. The methodology provides for the adoption of three constituent elements for the evaluation of the care dependency: (1) the clinical instability level of the patient (clinical stability dimension); (2) the patient’s ability to define his own needs and to choose the most suitable behaviors (responsiveness dimension); (3) the patient’s ability to act autonomously and effectively (independence dimension).

Sistema Informativo della Performance Infermieristica (SIPI): According to the health professionals’ interventions, SIPI [22] classifies the different healthcare profiles of the Health Operating Units representative of the care complexity. This information system allows the creation of a model for the processing of the evaluation form with nursing care data. In particular, this system aims to allocate healthcare personnel according to the complexity of nursing care, document the use of the nursing resource, monitor and determine the healthcare costs and define a distribution coefficient of support staff in relation to the care complexity.

The SIPI uses a detection form providing a concise and exhaustive frame for the assistance’s complexity profiles.

Indice di Complessità Assistenziale (I.C.A.): I.C.A. methodology was developed by Cavaliere [18] in 1999 on the basis of the “Model of Nursing Services”. This indicator shows both what are the characteristics of standardized health problems and possible variations. The aim is to direct the healthcare professional’s activity towards the identification of individual or general healthcare priorities (of the individual user or of a healthcare operating unit or even of entire healthcare organizations), thus guaranteeing continuous evaluation and improvement of quality. The I.C.A. allows both to define an evaluation form of the health professionals’ skills and to measure the qualitative and quantitative complexity of the interventions required by the case (intrinsic complexity of the health service). Healthcare professionals obtain the complexity measurement for each patient daily, through an objective, verifiable and reproducible measurement system.

## 2. Rationale of the Study

In the healthcare organizations, the allocation of patients in the different levels of care takes place exclusively on the basis of clinical, logistical and organizational evaluations, mainly linked to the type of pathology. On the other hand, in the literature, it is widely accepted that the level of intensity of care necessary to respond to the patient’s clinical and care needs depends on the integration of two dimensions: clinical instability and care dependence.

The concept of care dependence is defined as “a concept that includes not only the assessment of the physical, educational, relational needs of the patient or group of patients that the healthcare professional must face daily, but also the analysis of the activities and the context in which they are provided” [23]. The clinical instability’s concept is identified “with the severity and frequency of clinical disorders, in other words, with comorbidity or with other criteria received from acute disease” [24]. Care dependence and clinical instability determine the complexity of care. There are different care complexities for any level of intensity. They are two distinct elements and are always co-present in the patient. Clinical instability and care dependence may coincide perfectly, but in many other cases, they may not coincide [25]. The non-coincidence can lead to a contrast between the allocation of patients and the care tasks.

In this study, it was considered necessary to introduce valid and reliable methods for the evaluation of the two dimensions. The research aimed to take a “screenshot” of the care intensity in the various medical area settings and subsequently introduce the clinical-care complexity evaluation in a systematic way.

## 3. Objectives

The primary objective was to determine the intensity of care profiles (clinical instability + care dependence) of the patients allocated in the different settings of the medical area.

The secondary objectives were as follows:

- Verify the validity and reliability of the instruments chosen for measuring clinical severity and care dependence;
- Introduce the routine use of care intensity assessment tools to guide patient allocation.

## 4. Materials and Methods

The phases of the study were the following:

- Training meetings with health experts for the use of National Early Warning Score (NEWS) [26,27,28,29] and Index of Caring Dependency (ICD) [30,31] tools;
- Training meetings for the drafting and implementation of the NEWS form in clinical care practice;
- Systematic monitoring of the clinical instability level (NEWS) and care dependency (ICD);
- Retrospective analysis of public records relating to hospitalizations between 15 January and 15 February 2020.

### 4.1. Study Design

In order to evaluate the care complexity (clinical instability + care dependence) used in the different medical settings of the Italian healthcare organizations, a longitudinal retrospective study was conducted. This study was performed in a middle-size hospital located in the center of Italy. The hospital has a total of 433 beds. As for the three wards investigated in the study, Internal Medicine has 45 beds, Neurology has 14 beds and Pneumology has 14 beds.

The Internal Medicine ward deals with the diagnosis and treatment from a medical and non-surgical viewpoint of very complex and different pathologies such as infections, cardiovascular diseases, gastrointestinal diseases, blood diseases, etc.

The Neurology ward deals with the treatment of central and peripheral nervous system disorders. The most frequently treated diseases are headaches and other forms of headache, speech and movement disorders, infections of the brain and peripheral nervous system, stroke, neurodegenerative diseases, such as Alzheimer’s, Parkinson’s and amyotrophic lateral sclerosis.

The Pneumology ward deals with the diagnosis, therapy and follow-up of respiratory diseases in adults and children, from the most common to the most complex, such as bronchitis, flu, asthma, rhinitis, respiratory failure, chronic obstructive pulmonary disease, tuberculosis and others.

The tools identified by the Triage-in-the-Corridor (Tri-Co) [32] method used were the NEWS and the modified ICD (mICD).

The reference variables are those collected from public health records. An analysis “per day” was carried out for the organizational assessments, comparing the days of hospitalization for each subject, for each level of care intensity and for each different care settings.

For the validation of the instruments, a “per patient” analysis was used, comparing the levels of complexity identified at the entrance with external indices such as mortality, transfer to intensive care, hospitalization duration and economic value of the Diagnosis Related Group (DRG).

### 4.2. Inclusion Criteria

No identifiable human data were used for this study. All the records that anonymously identified subjects who were present in the hospital wards, neither discharged nor transferred during the period 15 January–15 February 2020 were included in the analysis. This pre-COVID19 time period was chosen because it was recommended for its highest number of hospital admissions by both physicians and healthcare managers. It was considered crucial in getting a balanced view of how organizational choices are made for the allocation of patients.

Instead, patients admitted or transferred on entry from another hospital ward outside the analyzed area were excluded as well as day hospital and outpatient patients.

A form for each record contained in the public database has been completed. The data collection was carried out by connecting online to the system.

### 4.3. Evaluation Tools

The choice of assessment tools was shared with healthcare professionals and was based on the following criteria: easy use, time required for compilation, applicability in context. For these reasons, the NEWS and mICD tools of the tri-co method already validated in this context were deemed suitable.

For study purposes, the admission value and the maximum value recorded on each day of hospitalization publicly reported in the online system were noted. Instead, the care dependency level (mICD) was assessed upon admission and was re-evaluated for each transfer of care setting.

The main characteristics of the tools used are shown below.

#### 4.3.1. Clinical Instability Level (NEWS)

**Figure 2:**
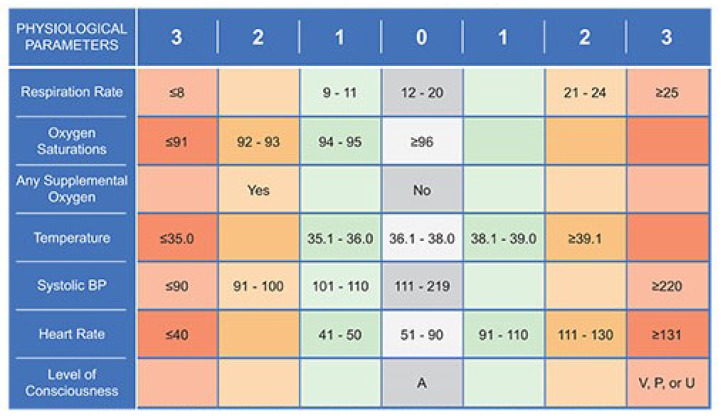
Form. Source: Jones [33]. The National Early Warning Score (NEWS) initiative from the Royal College of Physicians’ NEWSDIG was jointly developed and funded in collaboration with the Royal College of Physicians, Royal College of Nursing, National Outreach Forum and NHS Training for Innovation.

The NEWS scale was used to evaluate clinical instability, and this tool was recommended by the Tuscany region (Italy). The attribution of the evaluation scores of the clinical instability is based on 6 physiological parameters: systolic blood pressure, heart rate, respiration rate, oxygen saturation, temperature and level of consciousness (AVPU). Moreover, an additional score is provided for patients with oxygen therapy (Figure 2).

Heart rate: Heart rate measurement is an important indicator of clinical conditions. Tachycardia may indicate impaired circulation for sepsis, heart failure, fever, physical pain or other pathological conditions. It can also be due to cardiac arrhythmia, metabolic disorders (hyperthyroidism) or drug intoxication (sympathomimetic or anticholinergic). Bradycardia is equally important: a low rate can be normal in certain conditions or it can be a drugs consequence (beta blockers). However, it can be an important indicator of hypothermia, alteration of the central nervous system, hypothyroidism or heart block.

Respiration rate: High breathing frequency is an evident sign of acute disease and severe dysfunction. The frequency of breathing can also increase due to the physical pain felt, the states of anxiety, severe lung infection and serious metabolic alterations (for example, metabolic acidosis). A reduced breathing frequency is an important indicator of narcosis alteration.

Oxygen saturation: The non-invasive measurement of oxygen saturation is commonly used in acute conditions evaluation and the measurement technology is now easy to use and low cost. Being a relevant parameter for the evaluation of lung and cardiac function, it is a common component in the evaluation of the acute patients’ severity.

Temperature: Hyperthermia and hypothermia are detected in the NEWS system considering that extreme body temperature values are sensitive indicators of physiological damage.

AVPU: AVPU expresses the patient’s consciousness status. This evaluation is carried out in sequence. Alert: the patient is perfectly awake. Verbal: the patient responds in some way to the verbal warning, either by moving his eyes, by making sounds or by moving. Pain: the patient reacts to the painful stimulus. A patient who is not awake and who does not respond to the verbal warning can react to the painful stimulus by moving away from the pain source or with a response from the limbs. Unresponsive: at this stage the patient does not respond to verbal or painful stimuli and is therefore completely unconscious.

Supplemental oxygen: Patients who require supplemental oxygen to maintain saturation at acceptable levels are considered to be at higher risk. This dimension was considered fundamental and for this reason introduced in the NEWS form.

#### 4.3.2. Care Dependence (mICD)

For the evaluation of care dependence, the modified Index of Caring Dependence (mICD) was chosen. The original instrument included the evaluation of 7 care dimensions: nutrition/hydration, elimination, hygiene and comfort, mobilization, diagnostic procedures, therapeutic procedures and sensory perception. This tool has been modified by adding the dimension relating to the skin integrity as health workers have shown that this type of activity was not adequately represented in the tool. For each dimension, a choice between four levels of assistance was made (Table 1).

For each item, a score from 1 to 4 is assigned (range score 8–32), and the level of dependence on 3 levels is classified as follows:

- High care dependence, range score 24–32;
- Average care dependence, range score 15–23;
- Low care dependence, range score 8–14;

The matrix integration of levels of care dependence and clinical instability provides the synthetic Index of Caring Complexity (ICC). Higher scores represent a higher caring complexity (Table 2).

### 4.4. Analysis

The consistency level between the setting assigned to the patient and the care complexity resulting from the integration of NEWS and mICD was the main result. It is hypothesized that a high ICC reflects that the patient is allocated in settings with greater care dependence while the low care level should be reserved for patients with a lower level of clinical instability. The connection between the two instruments was assessed by creating ICC.

To carry out the statistical analysis, the STATA version 14.2 software (StataCorp LLC, College Station, TX, USA) was used. The main dispersion measures were calculated (mean, standard deviation, and 95% confidence intervals (CI)). The Shapiro–Wilk test was the approach chosen to testing normality. In this case, it is the most appropriate since can be used with an approximation accurate for 4 ≤ n ≤ 2000 [35].

Then, the Kruskal–Wallis H test (also called the one-way ANOVA on ranks), a rank-based nonparametric test used to determine if there are statistically significant differences between two or more groups of an independent variable on a continuous or ordinal dependent variable, was adopted.

## 5. Results

### 5.1. Study Population

In the period 15 January – 15 February 2020, 450 records, evaluated for 2884 hospitalization days and for each inpatient bed, were evaluated.

Furthermore, in order to test the assumption of normality and homogeneity of the considered variables, a Shapiro–Wilk test was performed. It revealed a non-normal distribution (*p* = 0.000). Then, a Kruskal–Wallis H test was conducted to determine if the age distribution was different for three hospital wards: (a) Internal medicine (*n* = 349), (b) Neurology (*n* = 39) and (c) Pneumology (*n* = 62). The Kruskal–Wallis H test showed that there was a statistically significant difference in patients’ age between the three groups, χ^2^(2) = 20.401, *p* = 0.0001.

The same nonparametric test was carried out to determine if the age distribution was different between the four-setting care complexity levels. The Kruskal–Wallis H test showed that there was a statistically significant difference in patients’ age between the four groups, χ^2^(3) = 60.882, *p* = 0.0001.

Thus, the average age of patients diverges both between the different hospital wards and between the different setting care complexity levels, with subjects significantly younger in the neurology ward and with more advanced age in low care (Table 3 and Table 4).

The characteristics of the sample are shown in Table 5. From the anonymous records accessible from the National Health Service platform, it can be seen that only patients from the Internal Medicine ward are hospitalized in the Low Care.

As for the outcome, Table 6 shows a poor “exchange” of patients within neurology ward (7.7%), while within the internal medicine ward it is equal to 14.9%.

### 5.2. Level of Care Complexity by Settings

**Figure 3:**
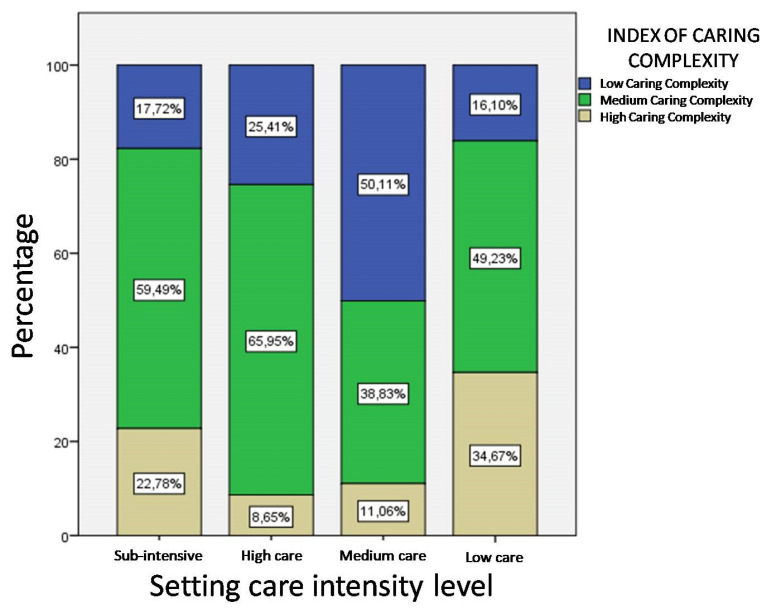
Distribution of care complexity levels on hospitalization days by setting care.

As shown in Table 7 and Table 8 and Figure 3, within the various settings, the distribution of the care complexity levels is not consistent with the care dependence level to which the patient was assigned. Moreover, in Table 7, it is possible to note that a high percentage of unstable patients are in the low care setting (19.5% and 16.8% respectively).

Most of the patients with a high level of care dependence, 153 subjects, are allocated in the medium setting level even if considering their health status, they would need more constant treatment (Table 8).

Ultimately, the proportion of patients with high care complexity (34.67%) found in low care is significantly higher than the other care settings, while only 16.1% of patients received a low level of care complexity consistent with patient allocation (χ^2^(4) = 6.289, *p*< 0.01) (Figure 3).

### 5.3. Level of Care Complexity by Hospital Wards

**Figure 4:**
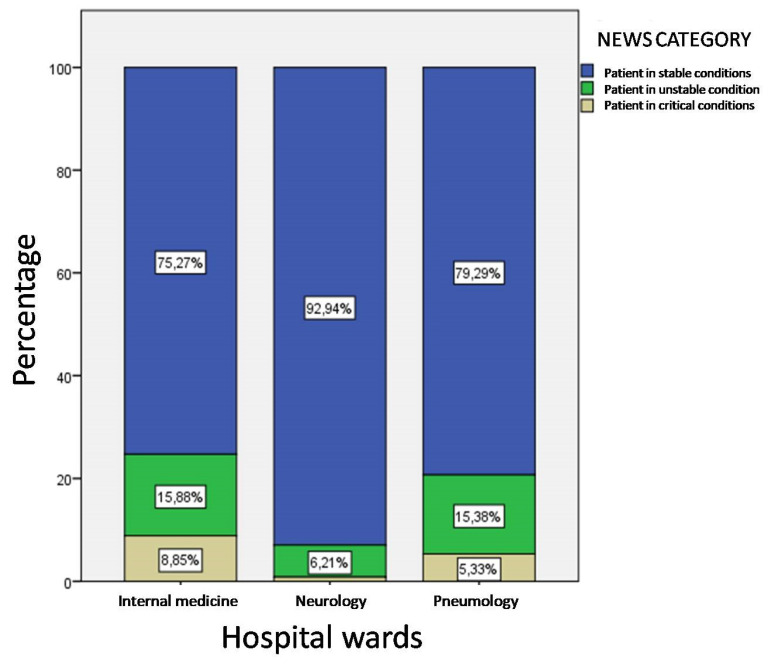
Distribution of clinical instability levels by hospital wards.

The distribution of NEWS scores between the three hospital wards (Figure 4) shows that the highest prevalence of patients in critical condition and with clinical instability is recorded among the patients belonging to the internal medicine ward (about 25%). The difference between the three hospital wards generated by patients in stable, unstable and critical conditions has achieved little significance (χ^2^(4) = 2.289, *p* = 0.06). Moreover, it should be noted that more than 90% of patients in neurology have conditions of clinical stability despite a 30% use of the medium care setting level.

**Figure 5:**
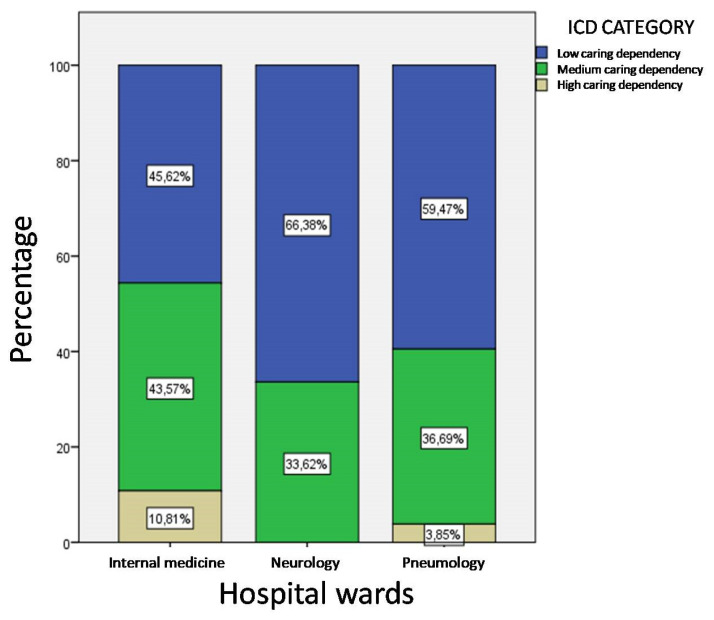
Distribution of levels of care dependence by hospital wards.

Taking care dependence into consideration, the difference with the Neurology ward is more evident, as it does not present even one observation with mICD > 23 and over two thirds of patients have low dependence on care (χ^2^(4) = 3.441, *p =* 0.04) (Figure 5).

**Figure 6:**
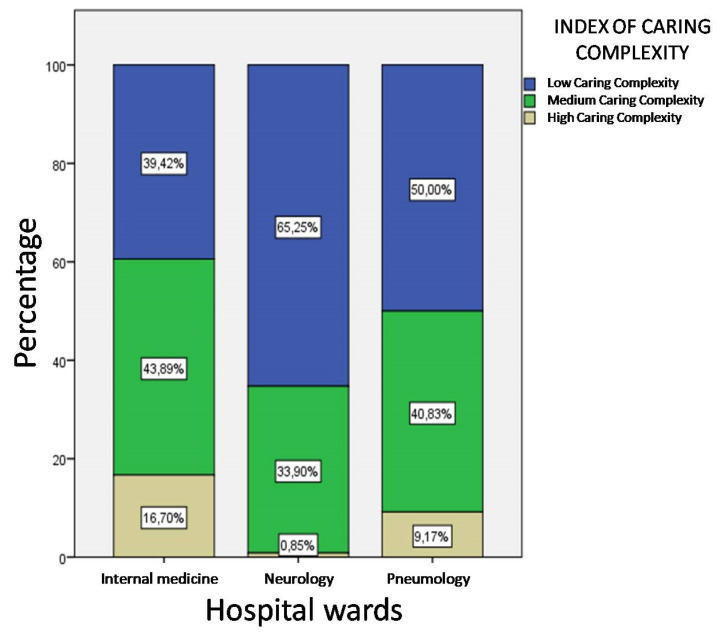
Distribution of care complexity levels by hospital wards.

Completely overlapping values are obtained by analyzing the ICC (Figure 6). The index shows that only a small proportion of observations (0.85%) highlighted the need for high care complexity among patients allocated in the neurology ward (χ^2^(4) = 5.935, *p* = 0.05).

## 6. Discussion

The allocation of patients with high clinical instability level and high care dependence was consistent with the healthcare organizational model that identifies the appropriate care complexity level for patients hospitalized in the high and sub-intensive care setting. Instead, the allocation of patients with medium and low care complexity is absolutely inadequate. Such a result is comparable with that obtained by Spagnolli et al., in which the implementation of an organizational model for intensity of medical care was associated with a decreasing trend in mortality over the six-year period [36].

In this work, the data investigated at the low care setting is paradoxical, since over a third of the patients were found to be highly care complex. The result is higher compared to the sub-intensive care setting where the provided healthcare assistance is two times greater than in the high care setting. This may be explained by the fact that frequently patients in the high care setting are allocated although they do not need immediate diagnostic and therapeutic interventions and only await transfer or discharge.

The lack of objective and standard criteria for patient admission within the high care setting means that the patient allocation in this setting takes place only for logistical organizational choices and not for choices deriving from the level of care intensity. Direct hospitalization in low care setting is often used when, in overcrowded conditions, inpatient beds are not available in other hospital wards. A previous study carried out in 2006 showed a positive influence of a model introduced for evaluating intensity of medical care. Specifically, better results, according to a multivariate logistic regression model, were mainly associated with a reduction in urgent transfers to intensive care units [37].

Another peculiar aspect of the present study is the different distribution of care tasks among the different hospital wards. In particular, patients hospitalized in the neurology ward have a very low level of complexity of care (ICC) when compared with patients hospitalized in the other two wards (Internal medicine and Pneumology). In addition, patients hospitalized in the Neurology ward present stable clinical conditions. This result can surely surprise. Generally, in neurological wards, patients with strokes are primarily hospitalized. These are often patients in life-threatening condition with limited mobility and often cardiovascular instability; they require advanced care from doctors, nurses and physiotherapists. Neurological ward patients are usually older. However, in the present case, the low level of complexity of care in this ward can be explained by the fact that many young patients with very serious pathologies but in conditions of clinical stability are hospitalized here. In addition, the average age of patients hospitalized in neurology (71.9) is significantly lower than in the other two wards (80.2 in Internal medicine and 75.3 in Pneumology). This causes a low level of healthcare assistance towards this type of patient, for whom there is no possibility of using the low care setting. However, Micieli et al. [38] have already highlighted how the healthcare-based model of intensity may be inadequate for the complex area of Neurology ward. Indeed, a limitation related to the implementation of this managerial model could lead to the loss of the multidisciplinary and multi-professional integration.

## 7. Conclusions

The “screenshot” of the organizational healthcare context realized in this study made it possible to highlight the aforementioned organizational problems and to envisage improvements to overcome the problems. In particular, new, more rigid and objective criteria for allocating patients in the low care setting have been defined. In addition, an advantage of the study was the introduction of the NEWS form in daily health practice. NEWS scores are used as a guiding criterion for allocating patients in the high care setting. At the same time, integrating the low care-staff with additional socio-health operators was considered a fundamental solution. Moreover, it was assessed and considered correct to use the low care setting also for patients hospitalized in the Pneumology ward in addition to those hospitalized in the Neurology ward who mainly foresee low care tasks.

Although this study provides innovative and diverse proposals for improvement the management and evaluation model of the patient care dependence, some limitations need to be addressed.

Firstly, the use of online data may have led to an underestimation of the real problem faced by healthcare organizations. This problem arises from the fact that often the updating of data in information systems is lacking, and only what is explicitly required by law is published. Secondly, the use of data available only for some hospital wards (Internal medicine, Pneumology and Neurology) does not allow validating the effectiveness and efficiency of the NEWS and mICD tools for the healthcare structure as a whole but allows to generalize them for those wards.

It is possible to conclude that the prospective use of standardized tools for assessing care complexity can represent a valid support for improving the organizational appropriateness of healthcare organizations.

## Figures and Tables

**Table 1:** Modified index of caring complexity with eight care dimensions. Source: Piu [34].

	mICD								
**Care Levels**		**Nutrition/Hydration**	**Elimination**	**Hygiene and Comfort**	**Mobilization**	**Diagnostic Procedures**	**Therapeutic Procedures**	**Sensory Perception**	**Skin Integrity**
	1	Independent	Independent	Independent	Independent	Monitoring once a day	Oral therapy only or no therapy	Patient alert and oriented (no sedatives)	Patient with low risk of pressure ulcers (PU) (Braden > 16). Absence PU
	2	Help nutrition	Bladder/condom catheter	Intimate hygiene in bed but independent in the use of services	Walk with help	Vital parameters’ monitoring once per shift	Oral, i.m., e.v.	Occasional temporal-space disorientation (day and night sedatives)	Patient at risk of PU (Braden ≤ 16) not bedridden with mobilization program
	3	Fed patient	Occasional urinary and fecal incontinence	Hygiene in bed with patient help	Armchair mobilization	Vital parameters’ monitoring more than once per shift	Central Venous Catheter with non-continuous infusion	Constant disorientation temporal-space (sedatives day and night)	Bedridden patient with mobilization program and/or presence of PU up to the 2nd degree
	4	Total parental nutrition	Permanent urinary and fecal incontinence	Hygiene in bed without patient’s help	Bedridden	Constant vital parameters’ monitoring	Central Venous Catheter continuous/infusion 24 h	Sleepy state, coma or severe state of agitation (delirium)	Bedridden patient with mobilization program and/or presence of PU >2nd degree

**Table 2:** Index of caring complexity (ICC) (clinical instability + care dependence).

ICC			
NEWS	ICD 8–14 0	ICD 15–23 1	ICD 24–32 2
NEWS 0–4 0	Low	Medium	High
NEWS 5–6 1	Medium	Medium	High
NEWS ≥7 2	High	High	High

**Table 3:** Average age by hospital wards.

Age				
Hospital Wards	Average	N	Std. Deviation	95% CI
Internal medicine	80.23	349	12.250	79.47–80.52
Neurology	71.95	39	13.594	71.5–72.33
Pneumology	75.27	62	13.823	75.16–75.68
Total	78.83	450	12.856	78.23–79.11

**Table 4:** Average age by setting.

Age				
Setting Care Complexity Level	Average	N	Std. Deviation	95% CI
Sub-intensive	72	17	15.576	71.84–72.16
High care	80.39	23	11.696	80.2–80.56
Medium care	78.69	361	12.911	78.4–78.78
Low care	81.47	49	11.288	81.32–81.91
Total	78.83	450	12.856	78.51–79.12

**Table 5:** Contingency table on the sample characteristic by hospital ward.

Sample Characteristics		Hospital Wards						Significance of Chi-Square
		Internal Medicine		Neurology		Pneumology		
		N	%	N	%	N	%	
Gender	Male	173	49.6	13	33.3	30	48.4	*p* < 0.1
	Female	176	50.4	26	66.7	32	51.6	
Age classes	<60	24	6.9	9	23.1	6	9.7	*p* < 0.001
	61–70	41	11.7	6	15.4	6	9.7	
	71–80	81	23.2	13	33.3	29	46.8	
	81–90	139	39.8	10	25.6	15	24.2	
	>90	64	18.3	1	2.6	6	9.7	
Setting care complexity level	Sub-intensive	11	3.2	1	2.6	5	8.1	*p* < 0.05
	High care	10	2.9	5	12.8	8	12.9	
	Medium care	280	80.2	33	84.6	49	79	
	Low care	48	13.8	0	0	0	0	
Hospitalization days	1–3	75	21.5	0	0	14	22.6	*p* < 0.001
	4–9	194	55.6	24	61.5	39	62.9	
	10–15	62	17.8	6	15.4	7	11.3	
	>15	18	5.2	9	23.1	2	3.2	

**Table 6:** Health outcome by hospital ward.

Health Outcome	Hospital Wards								Significance of Chi-Square
	Internal Medicine		Neurology		Pneumology		Total		
	N	%	N	%	N	%	N	%	
Ordinary discharge	239	68.5	28	71.8	50	80.6	317	70.4	*p* < 0.001
Transfer to another ward	7	2	2	5.1	1	1.6	10	2.2	
Death	40	11.50	0	0.00	1	1.60	41	9.10	
Re-entry	0	0.00	0	0.00	0	0.00	0	0.00	
Other institute	6	1.7	4	10.30	0	0.00	10	2.20	
Hospice	2	0.6	2	5.10	1	1.60	5	1.10	
Integrated home care	3	0.9	0	0.00	0	0.00	3	0.7	
Transfer to higher care level	11	3.2	0	0.00	2	3.2	13	2.9	
Transfer to lower care level	41	11.7	3	7.7	7	11.3	51	11.3	
Total	369	100.00	39	100.00	62	100.00	450	100.00	

**Table 7:** Distribution of clinical instability levels by setting care.

Setting Care Complexity Level	Clinical Instability Level (NEWS)						Significance of Chi-Square
	Stable Patient (Score 1–4)		Unstable Patient (Score 5–6)		Critical Conditions (Score ≥ 7)		
	N.	%	N.	%	N.	%	
Sub-intensive	31	39.2	35	44.3	13	17.6	*p* < 0.05
High care	123	66.5	55	29.7	7	3.8	
Medium care	1888	82.2	269	11.7	140	6.1	
Low care	205	63.5	67	19.5	55	16.8	
Total	2247	77.9	422	14.6	215	7.5	

**Table 8:** Distribution of levels of care dependence by setting care.

Setting Care Complexity Level	Care Dependence Levels (mICD)						Significance of Chi-Square
	Low (Score 8–16)		Medium (Score 17–25)		High (Score ≥ 26)		
	N.	%	N.	%	N.	%	
Sub-intensive	30	38.0	44	55.7	5	6.3	*p* < 0.05
High care	59	31.9	117	63.2	9	4.9	
Medium care	1289	56.1	855	37.2	153	6.7	
Low care	58	18.0	182	56.3	83	25.7	
Total	1436	49.8	1198	41.5	250	8.7	
